# *Rickettsia* spp. in bats of Romania: high prevalence of *Rickettsia monacensis* in two insectivorous bat species

**DOI:** 10.1186/s13071-021-04592-x

**Published:** 2021-02-10

**Authors:** Ioana A. Matei, Alexandra Corduneanu, Attila D. Sándor, Angela Monica Ionică, Luciana Panait, Zsuzsa Kalmár, Talida Ivan, Ionel Papuc, Cosmina Bouari, Nicodim Fit, Andrei Daniel Mihalca

**Affiliations:** 1 Department of Microbiology, Immunology and Epidemiology, Faculty of Veterinary Medicine, University of Agricultural Sciences and Veterinary Medicine Cluj-Napoca, Cluj-Napoca, Romania; 2 Department of Parasitology and Parasitic Diseases, Faculty of Veterinary Medicine, University of Agricultural Sciences and Veterinary Medicine Cluj-Napoca, Cluj-Napoca, Romania; 3 Department of Parasitology and Zoology, University of Veterinary Medicine, Budapest, Hungary; 4 Regele Mihai I al României” Life Science Institute, University of Agricultural Sciences and Veterinary Medicine Cluj-Napoca, Cluj-Napoca, Romania; 5 Department of Semiology, Faculty of Veterinary Medicine, University of Agricultural Sciences and Veterinary Medicine, Cluj-Napoca, Romania

**Keywords:** Chiroptera, Insectivorous bats, SFG rickettsiae, Vector-borne diseases, Zoonotic reservoir

## Abstract

**Background:**

Spotted fever group rickettsiae represent one of the most diverse groups of vector-borne bacteria, with several human pathogenic species showing an emerging trend worldwide. Most species are vectored by ticks (Ixodidae), with many zoonotic reservoir species among most terrestrial vertebrate groups. While the reservoir competence of many different vertebrate species is well known (e.g. birds, rodents and dogs), studies on insectivorous bats have been rarely performed despite their high species diversity, ubiquitous urban presence and importance in harboring zoonotic disease agents. Romania has a high diversity and ubiquity of bats. Moreover, seven out of eight SFG rickettsiae species with zoonotic potential were previously reported in Romania. Based on this, the aim of this study was to detect *Rickettsia* species in tissue samples in bats.

**Methods:**

Here we report a large-scale study (322 bats belonging to 20 species) on the presence of *Rickettsia* spp. in Romanian bat species. Tissue samples from insectivorous bats were tested for the presence of *Rickettsia* DNA using PCR detection amplifying a 381 bp fragment of the *gltA* gene. Positive results were sequenced to confirm the results. The obtained results were statistically analyzed by chi-squared independence test.

**Results:**

Positive results were obtained in 14.6% of bat samples. Sequence analysis confirmed the presence of *R. monacensis* in two bat species (*Nyctalus noctula* and *Pipistrellus pipistrellus*) in two locations.

**Conclusion:**

This study provides the first evidence of a possible involvement of these bat species in the epidemiology of *Rickettsia* spp., highlighting the importance of bats in natural cycles of these vector-borne pathogens.

**Graphical Abstract:**

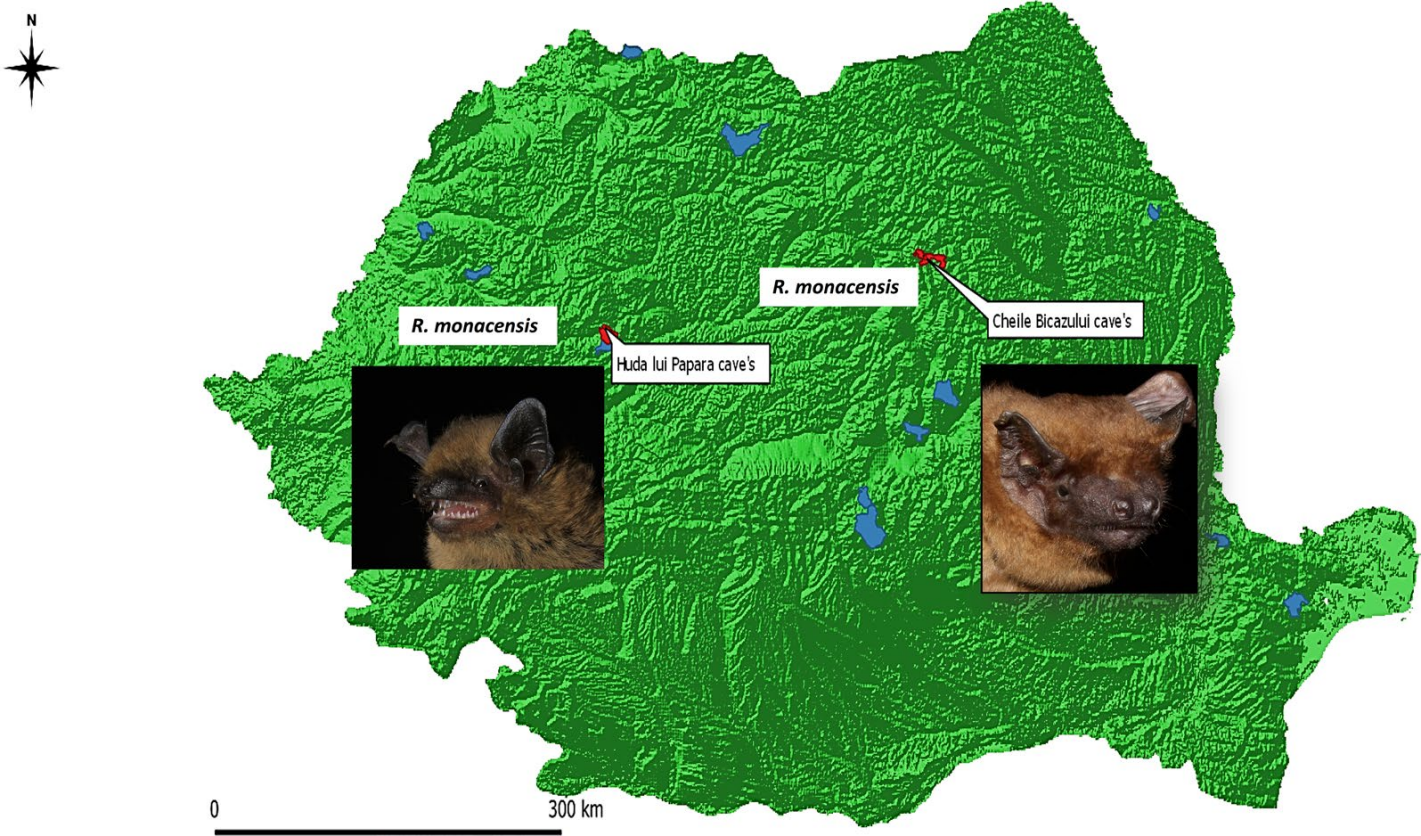

## Background

Among tick-borne diseases, rickettsioses are considered to be the oldest known affecting humanity. Tick-borne rickettsioses are caused by obligate intracellular bacteria belonging to the spotted fever group (SFG) of the genus *Rickettsia*, one of the most diverse groups among Rickettsiales, comprising a large number of zoonotic agents [1]. The first clinical description of the prototypical tick-borne rickettsiosis (Rocky Mountain spotted fever, RMSF) was made in 1899 by Edward E. Maxey [2]. Its agent, *Rickettsia rickettsii*, was demonstrated to have vectorial transmission in 1906 by Howard T. Ricketts [3], which was later confirmed by S. Burt Wolbach [4].

This complex of diseases is still intensively studied, and newly associated clinical conditions are continuously described. Its importance and recognition have increased considerably over the last 3 decades [1]. More than 24 *Rickettsia* species and subspecies are associated with human infections, while for many others the public health relevance is still unknown [1]. In Europe, eight tick-borne rickettsiae with known pathogenicity to humans were reported in European patients: *R. aeschlimannii*, *R. conorii* subsp. *conorii*, *R*. *helvetica*, *R*. *massiliae*, *R*. *monacensis*, *R*. *raoultii*, *R*. *sibirica* subsp. *mongolitimonae*, and *R*. *slovaca*. Additionally, 15 *Candidatus* Rickettsia species and strains of unknown pathogenicity were also described [1]. Seven of the eight human pathogenic species were previously reported in Romania (Table 1) [5–15]. Despite the common detection of tick-borne rickettsiae in ticks collected from diverse hosts, to the best of our knowledge, they have been reported only in humans and birds in Romania.

**Table 1:** Review of SFG Rickettsia detected in Romania

Rickettsia species	Organism	Ticks origin	References
*R. aeschlimannii*	*Hyalomma marginatum*	Cattle	[5]
*R. conorii*	humans	-	[6, 7]
	*Rhipicephalus sanguineus*	Dog	[8]
*R. helvetica*	*Ixodes ricinus*	Questing	[9]
	*I. ricinus*	Dogs, cattle, horses	[8, 10, 11]
	*I. ricinus*	Humans	[12]
	*I. ricinus*	*Erithacus rubecula*, *Panurus biarmicus*, *Turdus merula*	[13]
	*I. arboricola* *I. redikorzevi*	Birds*	[13]
	*I. ricinus* *I. crenulatus* *Dermacentor reticulatus* *R. sanguineus*	Dogs, foxes, sheep, cats	[5]**
*R. massiliae*	humans	-	[14]
	*I. ricinus* *I. arboricola*	*T. philomelos*	[13]
	*D. reticulatus*	Dogs, foxes	[5]
*R*. *monacensis*	*I*. *ricinus*	Questing	[9]
	*I*. *ricinus*	*E. rubecula*, *T. merula*	[13]
	*I*. *ricinus*	Dogs, cattle, horses	[8, 10]
	*I*. *ricinus*	Humans	[12]
	*Haemaphysalis concinna*	Birds*	[13]
	*I. ricinus* *D. reticulatus* *R. sanguineus*	Dogs, foxes, cats	[5]**
*R. raoultii*	humans	-	[14]
	*D. reticulatus*	Questing	[5, 9]
	*D. reticulatus*	Dogs	[8]
	*D. marginatus*	Cattle	[10]
	*D. reticulatus* *D. marginatus* *R. sanguineus*	Dogs, sheep, goats	[5]**
*R*. *slovaca*	humans	-	[14]
	*D. marginatus*	Cattle, sheep	[10]
	*D. reticulatus*	Dogs	[8]
	*I. ricinus*	*T. merula*	[13]
	*I. ricinus* *D. marginatus* *R. sanguineus*	Dogs, foxes, sheep, goats	[5]**

*The bird species is not specified

**Which tick species was collected from which host is not clearly specified

Romania has a remarkable biodiversity, having in its territory 21 ecoregions in 5 biogeographical regions [16]. This unique situation is associated with a wide range of habitats and is mirrored by a very rich fauna including 32 species of insectivorous bats [17], more than 82% of all continental European bat species [18]. Romania is not only diverse in species, but also hosts large bat populations, including two of the largest hibernating colonies of bats from Europe (> 100,000 individuals) [19].

Bats are well-known reservoir hosts for important zoonotic viruses such as *Lyssavirus* and *Ebola* virus*,* probable reservoirs for *Hendra* and *Nipah* henipaviruses, MERS and SARS coronaviruses, probably including SARS-CoV-2, and other emerging viruses [20, 21]. Alongside viruses, in the last few years, bacterial and protozoan pathogens from bats have been intensively studied to clarify their zoonotic potential. As a result, several pathogens have been identified in different samples (blood, tissues, ectoparasites, guano) such as *Babesia* spp. [22, 23], different strains of *Bartonella* spp. [24, 25], including species with zoonotic potential [26], *Borrelia* spp., some with a possible zoonotic potential [27, 28], and *Rickettsia* spp. [29, 30].

Bats are hosts for their specific ectoparasites such as bat bugs, flies, fleas, mites and ticks (hard and soft). In Europe, three species of hard ticks (*Ixodes ariadnae*, *I. simplex* and *I. vespertilionis*) and two species of soft tick (*Argas vespertilionis* and *A. transgariepinus*) are considered specific to bats [17]. However, other generalist tick species have been occasionally found on bats, including SFG-rickettsiae-vector ticks, such as *I. ricinus* and *Haemaphysalis concinna* [1, 17, 29], suggesting the possibility of harboring these pathogens.

Motivated by the high diversity and ubiquity of bats in Romania and their potential to harbor SFG rickettsiae, the aim of this study was to detect *Rickettsia* species in tissue samples in bats.

## Methods

### Tissue collection

The samples were collected from carcasses of bats accidentally killed, mainly by collisions (*n* = 165), or which died of natural causes (*n* = 157). The collection of carcasses was performed during all-year periods between 2011-2019. The majority of naturally caused deaths were recorded in the late spring. Bats were identified to the species level using morphological keys [31]. Some of the carcasses are found in the Grigore Antipa National Museum of Natural History in Bucharest. The carcasses were kept individually in plastic bags and deep frozen (– 20 °C) until analysis. A total of 322 bat samples belonging to 20 species (Additional file 1: Table S1) originating from 13 locations in Romania were necropsied. The heart tissue was used for DNA extraction, as this was the only tissue available from all the animals because of carcass damage (smashed, old, partially eaten, etc.). No individual animal was harmed or killed for the purpose of this study.

### DNA extraction

Genomic DNA was extracted from each tissue sample using commercial kits (ISOLATE II Genomic DNA Kit, Bioline, UK), following the manufacturer’s instructions. To assess cross-contamination between extracts, negative controls consisting only of reaction mixes were used. The DNA quantity and purity were assessed on a Nanodrop ND-1000 spectrophotometer analyzer (NanoDrop Technologies, Inc., Wilmington, DE, USA), using a representative number of randomly selected samples.

### Polymerase chain reaction (PCR) and agarose gel electrophoresis

The samples were assessed for the presence of SFG rickettsiae using a group-specific set of primers amplifying a 381 bp fragment of the rickettsial *gltA* gene (Rsfg877: GGGGGCCTGCTCACGGCGG; Rsfg1258: ATTGCAAAAAGTACAGTGAACA) [32]. The amplification was carried out in 25 µl reaction mixture containing 12.5 µl of Green PCR Master Mix (Rovalab GmBH), 6.5 µl PCR water, 1 µl of each primer (0.01 mM) and a 4 µl aliquot of isolated DNA. The amplification profile consisted of 5 min of initial denaturation at 95 °C, followed by 35 cycles of denaturation at 95 °C for 30 s, annealing at 53 °C for 30 s and extension at 72 °C for 30 s and a final extension at 72 °C for 5 min. In each PCR reaction set, positive and negative controls were included. Positive controls consisted of DNA extracted from *Ixodes ricinus* collected from a bird infected with *R. helvetica*, previously confirmed by sequencing (accession no. KR906075). The negative control consisted of reaction mix without DNA. The PCR was carried out using a T100^TM^ Thermal Cycler (Bio-Rad). PCR products were visualized by electrophoresis in a 1.5% agarose gel stained with SYBR^®^ Safe DNA gel stain (Invitrogen).

### DNA sequencing

All positive PCR products were purified using Isolate II PCR and Gel Kit (Bioline). Sequencing analysis was performed (Macrogen Europe, Amsterdam), and the obtained sequences were edited and analyzed using Geneious® (Biomatters LTD) 4.8.7 and compared with those available in the GenBank database by BLASTn analysis (http://blast.ncbi.nlm.nih.gov/Blast.cgi).

### Statistical analysis

The prevalence of *Rickettsia* spp. infection was calculated using Epi Info^TM^ 7 (CDC, USA) software with a 95% confidence interval. To evaluate differences in prevalence between localities and bat species, a chi-square test of independence was applied, and a *p*-value < 0.05 was considered significant.

## Results

SFG rickettsiae DNA was detected in 14.6% (*n* = 47/322, 95%CI: 11.02–19.04) of samples. Positive samples were detected in *Nyctalus noctula* (22 out of 188 tested), *Pipistrellus pipistrellus* (13 out of 55 tested), *Myotis alcathoe* (5 out of 12 tested), *P. nathusii* (4 out of 25 tested), *Plecotus auritus* (1 out of 6 tested), *Vespertilio murinus* (1 out of 4 tested) and *P. pygmaeus* (1 out of 2 tested) from five different locations (Table 2). The prevalence of *Rickettsia* spp. was 23.64% (95%CI: 13.23-37.02) in *P. pipistrellus* and 11.7% (95%CI: 7.48-17.18) in *N. noctula*. For the remaining species, *Rickettsia* spp. prevalence was not considered because of the small number of individuals tested.

**Table 2:** Presence of *Rickettsia* spp. DNA in bat species and their geographical origin (location/cave)

Species/location	Babadag	Bucureşti	Cheile Bicazului (cave)	Huda lui Papară Cave	Iaşi	Puciosu Mountain (cave)	Meziad Cave	Bat’s Cave, Braşov	Sfântu Gheorghe	Tulcea	Cluj	No. positive/no. tested
*Myotis alcathoe*	-	-	-	-	-	5/12	-	-	-	-	-	5/12
*Nyctalus noctula*	0/12	0/6	21^a^/104	1/11	0/51	-	0/1	-	0/1	0/1	0/1	22/188
*Pipistrellus nathusii*	4/25	-	-	-	-	-	-	-	-	-	-	4/25
*P. pipistrellus*	-	-	0/1	12^b^/53	-	-	-	1/1	-	-	-	13/55
*P. pygmaeus*	1/2	-	-	-	-	-	-	-	-	-	-	1/2
*Plecotus auritus*	-	-	-	-	-	1/6	-	-	-	-	-	1/6
*Vespertilio murinus*	-	0/1	-	-	-	1/2	-	-	-	-	0/1	1/4

^a^Twelve confirmed by sequencing of a 381 bp *gltA* gene fragment

^b^Five confirmed; -: no samples

0: no positive samples

Among the 47 positive samples, only 17 were suitable for sequencing (> 30 ng/μl DNA concentration). The sequences analysis showed 99.7% to 100% identity with *R. monacensis* found in *I. ricinus* from different geographical origins such as: Romania, Italy or Serbia (accession no. JX003686; GQ925822; KY203388). Four obtained sequences of *gltA* fragments (MT741493–MT741496) were deposited in the GenBank database. The short length of the sequences allowed the confirmation of the Rickettsia species, but they were inappropriate for further testing of strain genetic variability.

Following sequence analysis, *R. monacensis* presence was confirmed in all 17 sequences of bat samples, in 5 *P. pipistrellus* (accession no. MT741493-MT741494) and in 12 *N. noctula* (accession no. MT741495-MT741496).

All the confirmed positive samples originated from two distinct locations (Table 2). Since in these two locations these two species represented almost half of the collected samples (104 out of 188 *N. noctula* in Cheile Bicazului and 53 out of 55 *P. pipistrellus* from “Huda lui Papara” Cave, Additional file 1: Table S1), the differences in prevalence obtained for different locations or species were not significant.

## Discussion

To the best of our knowledge, this study is the first report of SFG rickettsiae in bat tissue samples in Europe and the first report of *R. monacensis* in tissues of *P. pipistrellus* and *N. noctula* (insectivorous bats) worldwide*.* Although SFG rickettsiae were not previously detected in bat tissue samples in Europe, SFG rickettsiae and related *Rickettsia* species were detected in bat specialist ectoparasites in Europe as well in other parts of the world (Table 3) [33–45]. This may suggest bat’s and/or their ectoparasites potential involvement in the transmission of these pathogens. This hypothesis is also sustained by the detection of SFG rickettsiae in bat tissue samples on other continents. For instance, in serological studies, antibodies against several *Rickettsia* spp. (*R. amblyommii, R. conorii, R. parkeri*, *R. rickettsia* and *R. rhipicephali*) were identified in bat samples collected from North and South America and from Asia [46–48]. Moreover, SFG *Rickettsia* spp. DNA was identified in blood or tissue samples from bats in Africa, America and Asia [49–51]. Different bat species (*Miniopterus natalensis*, *Nycteris thebaica*, *Epomophorus wahlbergi*, *Scotophilus dinganii* and *Glauconycteris variegata*) from South Africa were found positive for the presence of *Rickettsia* DNA, belonging to the SFG group, closely related to *R. conorii* [49]. In Saint Kitts, rickettsial DNA (99% similar to *R. africae* 17 kD protein gene) was detected in blood samples of *Artibeus jamaicensis* and *Ardops nichollsi* [50]. In China, *R. parkeri, R. lusitaniae, R. slovaca and R. raoultii* were detected in tissue samples collected from *P. pisterellus* [51]. In addition, *R. raoultii* and *R. rickettsii* were also detected in their ectoparasites (*A. vespertilionis*). Among these, four ticks positive for *R. raoultii* were removed from a *R. raoultii*-positive bat [49], suggesting the possible involvement of *A. vespertilionis* in the transmission of SFG rickettsiae among bats.

**Table 3:** *Rickettsia* spp. (SFG and RFG and related species) in bat specialist ectoparasites (soft ticks and other, specified in brackets)

Species	Host	Origin	Country	Ref.
*Candidatus* ‘Rickettsia andeanae’	*Trichobius joblingi* (fly)	*Carollia perspicillata*	Brazil	[33]
*Candidatus* ‘Rickettsia nicoyana’—related to *Candidatus* ‘Rickettsia wissemanii’ and *R. peacockii*	*O. knoxjonesi*	*B. plicata*	Costa Rica	[34]
*Candidatus* ‘Rickettsia wissemanii’—related to *R. peacockii*	*O. hasei*	*Noctilio albiventris*	French Guiana	[35]
*Candidatus* ‘Rickettsia wissemanii’	*O. hasei*	*Eptesicus diminutus*	Argentina	[36]
*Candidatus* ‘Rickettsia wissemanii’	*O. hasei*	*Artibeus planirostris*	Brazil	[37]
*R. africae*-like sequences	*A. vespertilionis*	*M. dasycneme*	Hungary	[38]
*R. helvetica*	*A. vespertilionis*	*V. murinus*	China	[38]
*R. helvetica*	*Nicteridopsylla eusaeca* (fly)	*N. noctula*	Hungary	[39]
*R. hoogstraalii*	*A. transgariepinus*	*P. hesperidus*	South Africa	[38]
*R. lusitaniae*	*O. yumatensis*	Caves walls	Mexico	[40]
*R. lusitaniae*	*Ornithodoros* spp.	*Balantiopteryx plicata*	Mexico	[38]
*R. lusitaniae*	*A. vespertilionis*	*V. murinus*	China	[38]
*Rickettsia* spp. Av 22 related to AvBat	*A. vespertilionis*	Multiple bats species	Hungary	[38]
*Rickettsia* spp. AvBat, related to *Rickettsia* sp. strain S and *R. africae*	*A. vespertilionis*	Home attic	France	[27]
*Rickettsia* spp. related to *R. honei*-like strains	*A. vespertilionis*	*Scotophilus kuhlii*	Pakistan	[41]
*Rickettsia* spp*.* related to *R. massiliae* and *Candidatus* ‘Rickettsia barbariae’	*Spinturnix myoti* (mite)	*M. myotis*	Poland	[42]
*Rickettsia* spp. related to *R. peacockii* and *R. rickettsii*	*Carios (O.) kelleyi*	buildings	Iowa, USA	[43]*
*Rickettsia* spp*.* related to *R. raoultii*	*Eucampsipoda madagascarensis* (fly) *Penicillidia leptothrinax* (fly)	*Rousettus madagascariensis*	Madagascar	[44]
*Rickettsia* spp. related to *R. sibirica* and *R. conorii* (17kDA 100% similar)	*A. vespertilionis*	*P. pipistrellus* *P. auritus*	England	[45]

^*^In this study it was also demonstrated that both trans-stadial and transovarial transmissions are possible

*Rickettsia monacensis* has a wide distribution, and has been detected all over Europe in its main vector, *I. ricinus* [reviewed by 1,52,53]. The prevalence of *R. monacensis* in ticks varied between 0.5% in Germany and 57% in Italy [reviewed by 1,53]. Currently *R. monacensis* is considered a zoonotic species, causing MSF-like illness in humans [54, 55]. Its presence in bat tissues observed in this study may suggest bats' involvement in *Rickettsia* spp. epidemiology also in Europe. Soft ticks collected from *P. pipistrellus* have already yielded five different *Rickettsia* spp. DNA (*Rickettsia* sp. AvBat [27], *R. sibirica* and *R. conorii*—[40], *R. lusitaniae* and *Rickettsia africae*-like—[38]), while fleas and feces collected from *N. noctula* tested positive for *Rickettsia* spp. in Hungary [30, 42]. In addition, *R. helvetica* was previously detected in its vector (*I. ricinus*) tick collected from bats (*Rhinolophus hipposideros* and *M. myotis*) from Poland [29]. Thus, either these two bat species regularly harbor *Rickettsia* spp. infections or their associated ectoparasites (especially the soft tick, *A. vespertilionis*) may play a vectorial role in these bacteria. *Argas vespertilionis* commonly infests these bats and also is known to bite humans [56]. In addition, *P. pipistrellus* and *N. noctula* species are the most common bats in urbanized areas, with important populations roosting in anthropogenic roosts [31], altogether making them of interest in the study of vector-borne pathogen cycles and highlighting their importance as possible candidates for *Rickettsia* spp. reservoirs in urbanized habitats.

## Conclusions

The detection of *R. monacensis* in the present study reconfirms the presence and circulation of this SFG rickettsiae in insectivorous bats. To the best of our knowledge, this study represents the first detection of *R. monacensis* in bat tissue samples in Europe and its first detection in *P. pipistrellus* and *N. noctula* bat species. Considering the zoonotic potential of *R. monacensis*, the detection of putative and also known vectors in bats and its detection in bat tissue samples, it is important to establish the importance of bats in the epidemiology of *Rickettsia* spp. in general, and *R. monacensis* in particular, by further research.

## Supplementary Information

**Additional file1: Table S1**. Number of bat samples included in the study according to the species and geographical origin (location/cave) (DOCX 21 KB)

## Data Availability

All data generated or analyzed during this study are included in this published article [and its supplementary information files].

## Abbreviations

Acc. no.: GeneBank accession number
DNA: Deoxyribonucleic acid
MSF: Mediterranean spotted fever
MERS: Middle East respiratory syndrome
PCR: Polymerase chain reaction
RFG: Rickettsia felis group
RMSF: Rocky Mountain Spotted Fever
SARS: Severe Acute Respiratory Syndrome
SFG: Spotted Fever Group
