# Publisher Correction to: *Rickettsia* spp. in bats of Romania: high prevalence of *Rickettsia monacensis* in two insectivorous bat species

**DOI:** 10.1186/s13071-021-04702-9

**Published:** 2021-04-06

**Authors:** Ioana A. Matei, Alexandra Corduneanu, Attila Sándor, Angela Monica Ionica, Luciana Panait, Zsuzsa Kalmár, Talida Ivan, Ionel Papuc, Cosmina Bouari, Nicodim Fit, Andrei Daniel Mihalca

**Affiliations:** 1 Department of Microbiology, Immunology and Epidemiology, Faculty of Veterinary Medicine, University of Agricultural Sciences and Veterinary Medicine Cluj-Napoca, Cluj-Napoca, Romania; 2 Department of Parasitology and Parasitic Diseases, Faculty of Veterinary Medicine, University of Agricultural Sciences and Veterinary Medicine Cluj-Napoca, Cluj-Napoca, Romania; 3 Department of Parasitology and Zoology, University of Veterinary Medicine, Budapest, Hungary; 4 Regele Mihai I al României” Life Science Institute, University of Agricultural Sciences and Veterinary Medicine Cluj-Napoca, Cluj-Napoca, Romania; 5 Department of Semiology, Faculty of Veterinary Medicine, University of Agricultural Sciences and Veterinary Medicine, Cluj-Napoca, Romania

## Publisher Correction to: Parasites Vectors (2021) 14:107 https://doi.org/10.1186/s13071-021-04592-x

Following publication of the original article [1], it was brought to our attention that the article’s graphical abstract had been erroneously included in the Abstract of the PDF.

The PDF in the original article has now been corrected. The publisher apologizes for any inconvenience caused by this technical error.
