# Supplementary material for: Rickettsia spp. in bats of Romania: high prevalence of Rickettsia monacensis in two insectivorous bat species
Source: Parasit Vectors. 2021 Feb 10;14:107. doi: 10.1186/s13071-021-04592-x (PMC7873661; doi:10.1186/s13071-021-04592-x)
Supplement: Supplementary file 1 — Additional file1: Table S1. Number of bat samples included in the study according to the species and geographical origin (location/cave) (DOCX 21 KB) [file 13071_2021_4592_MOESM1_ESM.docx]

Table S1: Number of bats’ samples according to the species of and their geographical origin (location/cave) included in the study.

| Location  Species | Babadag | Bucureşti | Cheile Bicazului (cave) | Huda lui Papară Cave | Iaşi | Puciosu Mountain (cave) | Valea Leşului -Wather Cave | Meziad Cave | Bat’s Cave, Braşov | Sfântu Gheorghe | Tăuşoarele Cave | Tulcea | Cluj | Total |
| --- | --- | --- | --- | --- | --- | --- | --- | --- | --- | --- | --- | --- | --- | --- |
| *Barbastella barbastellus* | - | - | - | - | - | 2 | - | - | - | - | - | - | - | 2 |
| *Eptesicus serotinus* | 1 | - | - | 1 | - | - | - | - | - | - | - | - | - | 2 |
| *Hypsugo savii* | 1 | - | - | - | - | - | - | - | - | - | - | - | - | 1 |
| *Miniopterus schreibersii* | - | - | - | 4 | - | - | - | 1 | - | - | - | - | - | 5 |
| *Myotis alcathoe* | - | - | - | - | - | 12 | - | - | - | - | - | - | - | 12 |
| *Myotis bechsteinii* | - | - | - | - | - | 3 | - | - | - | - | - | - | - | 3 |
| *Myotis branditti* | - | - | - | - | - | 3 | - | - | - | - | - | - | - | 3 |
| *Myotis myotis* | - | - | - | - | - | 3 | 1 | - | - | - | 1 | - | - | 5 |
| *Myotis mystacinus* | - | - | - | - | - | 1 | - | - | - | - | - | - | - | 1 |
| *Myotis nattererii* | - | - | - | - | - | 1 | - | - | - | - | - | - | - | 1 |
| *Nyctalus leisleri* | 3 | - | - | - | - | - | - | - | - | - | - | - | - | 3 |
| *Nyctalus noctula* | 12 | 6 | 104 | 11 | 51 | - | - | 1 | - | 1 | - | 1 | 1 | 188 |
| *Pipistrellus kuhlii* | 1 | - | - | - | - | - | - | - | - | 1 | - | - | - | 2 |
| *Pipistrellus nathusii* | 25 | - | - | - | - | - | - | - | - | - | - | - | - | 25 |
| *Pipistrellus pipistrellus* | - | - | 1 | 53 | - | - | - | - | 1 | - | - | - | - | 55 |
| *Pipistrellus pygmaeus* | 2 | - | - | - | - | - | - | - | - | - | - | - | - | 2 |
| *Pipistrellus* spp. | 1 | - | - | - | - | - | - | - | - | - | - | - | - | 1 |
| *Plecotus auritus* | - | - | - | - | - | 6 | - | - | - | - | - | - | - | 6 |
| *Rhinolophus ferrumequinum* | - | - | - | - | - | - | - | - | 1 | - | - | - | - | 1 |
| *Vespertilio murinus* | - | 1 | - | - | - | 2 | - | - | - | - | - | - | 1 | 4 |
| ***Total*** | 46 | 7 | 105 | 69 | 51 | 33 | 1 | 2 | 2 | 2 | 1 | 1 | 2 | 322 |
